# The relationship between job crafting and professional identity among primary and secondary school physical education teachers: a chain mediation analysis

**DOI:** 10.3389/fpsyg.2026.1864147

**Published:** 2026-06-18

**Authors:** Yangyang Fu, Tong Liu, Zhonggen Yin, Qiang Guo, Duan Yu, Li Yang, Mingda Li

**Affiliations:** 1 School of Sports Training, Chengdu Sport University, Chengdu, China; 2 College of Physical Education and Health Management, Chongqing University of Education, Chongqing, China

**Keywords:** job crafting, positive psychological capital, primary and secondary school physical education teachers, professional identity, work stress

## Abstract

**Background:**

Job crafting is a proactive behavior through which teachers adjust their work tasks, relationships, and cognitions, and is positively associated with professional identity. However, the psychological mechanism linking job crafting to professional identity remains unclear among primary and secondary school physical education (PE) teachers. This study investigated this relationship, focusing on the chain mediating roles of work stress and positive psychological capital.

**Methods:**

The sample consisted of 850 primary and secondary school physical education teachers from 10 provinces in China (50% male, 50% female; mean age 34.75 ± 8.42 years). They completed measures of job crafting, professional identity, work stress, and positive psychological capital. Mediation analysis was conducted using SPSS 26.0 with the PROCESS macro (Model 6), applying 5,000 bootstrap resamples.

**Results:**

Job crafting was significantly and positively correlated with professional identity (r = 0.433, *p* < 0.001). Regression results showed that job crafting positively predicted professional identity (*β* = 0.433, *p* < 0.001), negatively predicted work stress (*β* = −0.351, *p* < 0.001), and positively predicted positive psychological capital (*β* = 0.459, *p* < 0.001). Work stress negatively predicted professional identity (β = −0.453, *p* < 0.001), whereas positive psychological capital positively predicted it. More importantly, the indirect effects of job crafting on professional identity via work stress alone, via positive psychological capital alone, and via the sequential pathway of work stress followed by positive psychological capital were all significant (95% CI did not include zero). The total indirect effect accounted for 51.4% of the total effect.

**Conclusion:**

Job crafting is positively associated with professional identity among primary and secondary school physical education teachers, with work stress and positive psychological capital serving both independent and chain mediating roles. These findings highlight that reducing work stress and boosting positive psychological capital form a key psychological pathway through which job crafting is associated with higher levels of professional identity, offering practical value for designing targeted teacher development interventions.

## 1 Introduction

This research provides three novel contributions: (a) integrating job crafting, work stress, psychological capital, and professional identity into a single chain mediation model; (b) applying conservation of resources theory to PE teachers’ unique work context; (c) revealing a “stress reduction → resource accumulation” sequential mechanism not previously demonstrated. Job crafting, first introduced by Wrzesniewski and Dutton, refers to the proactive adjustments employees make to their work tasks, relationships, and perceptions, aiming to better align their jobs with personal skills, interests, and values (Wrzesniewski and Dutton, 2001). Although job crafting is generally seen as a positive strategy linked to work meaning and personal accomplishment, growing evidence suggests its forms and effects vary across different occupational groups (Hakanen et al., 2018). It is also important to distinguish job crafting from top-down job redesign initiated by organizations. Unlike the latter, job crafting is not mandated by the organization but represents a bottom-up, self-initiated behavior (Tims and Bakker, 2010). Nevertheless, excessive or inappropriate job crafting may also lead to role ambiguity or increased workload (Dierdorff and Jensen, 2018). Accordingly, this study focuses on the job crafting tendencies of primary and secondary school physical education teachers, rather than job redesign through organizational interventions.

Professional identity reflects teachers’ recognition and affirmation of the value, meaning, and roles of their profession, and plays a key role in their professional development (Steinert et al., 2019). As the main agents of school physical education, primary and secondary school PE teachers’ level of professional identity affects not only the quality of their instruction but also students’ physical fitness and the development of exercise habits. In real-world educational settings, PE teachers often face marginalization, low social status, and insufficient resources, which in turn is associated with a decline in their professional identity (Amorim and Ribeiro-Silva, 2022). A survey of primary and secondary school PE teachers in China found that their disadvantaged position in social status, career development, and resource allocation makes them prone to burnout, with professional identity showing a significant negative correlation with work stress (Li et al., 2025). Therefore, exploring how proactive individual behaviors are related to PE teachers’ professional identity holds both theoretical and practical significance.

Job crafting, as a proactive behavioral strategy for adapting to the work environment, has been shown to positively related to individuals’ sense of professional meaning and belonging (Wrzesniewski et al., 2013). Among teachers, job crafting helps meet various work challenges, alleviates burnout, and higher levels of professional identity (Zhai et al., 2025). However, the influence of job crafting on professional identity is not always direct or positive. For instance, some research finds that when teachers reshape their work in ways that exceed their own capabilities or organizational expectations, it may instead be associated with role conflict and increased perceived stress (Leana et al., 2009). This suggests the link between job crafting and professional identity may be indirect, operating through certain psychological mechanisms.

Psychological factors are likely at the heart of this mediating mechanism. Work stress refers to a state in which perceived job demands exceed one’s coping resources, and it correlates negatively with teachers’ professional identity (Kyriacou, 2001). Chronic high work stress is negatively related to teachers’ positive feelings toward their profession and their sense of its value. Positive psychological capital, encompassing self-efficacy, hope, optimism, and resilience, has been shown to buffer the negative effects of work stress and positively related to professional identity (Avey et al., 2009). Although previous studies have examined the relationships among job crafting, work stress, positive psychological capital, and professional identity separately, few have integrated them into a chain mediation model focusing specifically on primary and secondary school PE teachers. PE teachers face a unique work environment: they not only deliver physical education instruction but also organize extracurricular training and competitions, all while coping with pressures such as inadequate facilities and heavy safety responsibilities (Yang, 2026). Therefore, examining how job crafting is associated with professional identity through the chain mediation of work stress and positive psychological capital in this population can both enrich career development theory for PE teachers and provide empirical support for targeted interventions.

To summary, this study aims to examine the link between job crafting and professional identity among primary and secondary school PE teachers, with a focus on the independent and chain mediating roles of work stress and positive psychological capital.

## 2 Literature review and hypotheses

### 2.1 Job crafting and professional identity

Professional identity refers to an individual’s positive cognition, affect, and value evaluation of their chosen occupation, reflecting the extent to which they have internalized the professional role into their self-concept (Ibarra, 1999). Related concepts such as specialty identity and organizational identity exist, but each differs from professional identity. Specialty identity focuses on a sense of belonging to a particular professional field; organizational identity centers on attachment to one’s workplace; professional identity, by contrast, transcends specific positions or organizations, pointing to an overall acceptance of and commitment to the occupation itself (Lammers et al., 2013). This study focuses on the professional identity of primary and secondary school PE teachers, as it not only affects their teaching engagement, occupational well-being, and intention to stay but also indirectly shapes the quality of school physical education. Nevertheless, existing research indicates that these teachers generally experience low professional identity, manifested in frequent burnout, role ambiguity, and insufficient self-efficacy (Li and Wang, 2024). Against the backdrop of basic education reform and mounting pressures in school physical education, investigating the antecedents and underlying mechanisms that enhance professional identity among primary and secondary school PE teachers holds significant practical value.

Job crafting refers to the bottom-up behavioral changes employees proactively make to adjust their work tasks, work relationships, and perceptions of work, with the core idea being that individuals actively shape the meaning and boundaries of their jobs according to their own needs and abilities. According to job crafting theory, such crafting improves the fit between individuals and their jobs, enhances the sense of meaning and autonomy at work, and thereby positively influences occupational attitudes (Wrzesniewski and Dutton, 2001). For primary and secondary school PE teachers specifically, when they actively seek challenging content in teaching tasks, expand positive interactions with colleagues and students, and re-examine the educational value of physical education, they are more likely to experience the intrinsic rewards of the PE teaching profession, thereby strengthening their identification with it (Day et al., 2006). This reasoning is supported by empirical research, which shows that job crafting positively predicts teachers’ professional identity, operating indirectly through increased work engagement and reduced emotional exhaustion (Leana et al., 2009). Based on the above theoretical framework, this study proposes the following hypothesis:

*H1:* Job crafting is positively associated with professional identity among primary and secondary school physical education teachers.

### 2.2 The mediating role of work stress

Work stress refers to the negative emotional and physiological responses arising from a perceived imbalance between job demands and one’s own resources in the work environment (Dunnette and Hough, 1992). For primary and secondary school PE teachers, work stress stems mainly from multiple role conflicts, heavy teaching loads, responsibility for student safety, competition for professional title promotion, and the societal perception of physical education as a marginalized subject (Lammers et al., 2013). Chronic work stress not only harms PE teachers’ physical and mental health but also erodes their occupational enthusiasm and sense of belonging, thereby negatively affecting professional identity. Research indicates that Chinese primary and secondary school PE teachers generally experience high levels of occupational stress, closely linked to the combined effects of physical education curriculum reform, school-level performance assessment requirements, and parental expectations (Wang et al., 2025). Therefore, uncovering the mediating role of work stress between teachers’ proactive behaviors and occupational attitudes holds significant implications for designing effective intervention strategies.

According to conservation of resources theory, individuals tend to strive for acquiring, maintaining, and protecting the resources they value. When facing resource loss or an imbalance between resource investment and returns, stress arises, which in turn triggers a range of maladaptive occupational behaviors and attitudes (Hobfoll, 1989). As a proactive strategy for acquiring and managing resources, job crafting helps teachers redesign work tasks, expand social support networks, and reframe work meaning, thereby effectively alleviating or preventing the accumulation of work stress (Tims et al., 2013). Specifically, when PE teachers proactively adjust teaching content to match student interests, strengthen collaborative teaching research with colleagues, and re-examine the unique value of physical education in student development, they experience greater autonomy and competence, which in turn reduces stress reactions stemming from role ambiguity or heavy workloads (Day et al., 2006). Lowered work stress then fosters positive cognitions and emotional attachment to the profession, thereby associated with professional identity. Conversely, a lack of job crafting behaviors may leave teachers trapped in a passive mode of coping with work difficulties, leading to continuously elevated work stress and ultimately eroding professional identity. Based on this theoretical logic, the study proposes the following hypothesis:

*H2:* Work stress mediates the relationship between job crafting and professional identity among primary and secondary school physical education teachers.

### 2.3 The mediating role of positive psychological capital

Positive psychological capital refers to a positive psychological state individuals display during growth and development, typically comprising four core dimensions: self-efficacy, hope, optimism, and resilience (Luthans et al., 2006). Self-efficacy refers to an individual’s confidence in completing tasks within specific situations (Bandura, 1977); hope refers to goal-directed willpower and the ability to plan pathways (Snyder, 1994); optimism refers to positive attributions and expectations for current and future success (Avey et al., 2009); resilience refers to the ability to recover and even thrive when facing adversity and setbacks (Masten, 2001). Unlike relatively stable personality traits, positive psychological capital is state-like and developable, meaning it can be associated with through training and intervention (Luthans et al., 2010). For primary and secondary school PE teachers, positive psychological capital serves not only as a key psychological resource for combating burnout and associated with subjective well-being but also as a crucial internal factor for sustaining long-term career commitment and identification. However, existing research shows that when teachers face practical difficulties such as insufficient workplace support and low social status, their level of positive psychological capital tends to decline (Skaalvik and Skaalvik, 2017). Hence, exploring the mediating mechanism of positive psychological capital between teachers’ proactive behaviors and professional identity holds significant theoretical and practical value for intervention.

Drawing on positive organizational behavior theory, positive psychological capital positively predicts work attitudes and behaviors by strengthening individuals’ intrinsic motivation, coping capacity, and goal commitment (Luthans et al., 2016). Job crafting, as a proactive process of resource building and meaning making, effectively facilitates the accumulation of positive psychological capital (Uen et al., 2021). Concretely, when PE teachers proactively adjust their teaching tasks (e.g., designing innovative lessons), expand relational resources (e.g., building supportive interactions with colleagues and parents), and reframe work cognitions (e.g., re-appreciating physical education’s value for students’ holistic development), they experience more success and receive more positive feedback, which boosts self-efficacy. At the same time, the sense of autonomy and control generated by job crafting helps strengthen hope and optimism. When facing setbacks in teaching (e.g., students’ negative attitudes toward PE class), job crafting enables teachers to actively seek alternative approaches, thereby reinforcing resilience. Associated with positive psychological capital, in turn, fosters teachers’ sense of professional identity: those with higher self-efficacy are more confident in their ability to fulfill the PE teacher role; those with greater hope and optimism are more willing to commit to the profession over the long term; and those with higher resilience are more likely to see career challenges as opportunities for growth rather than threats. Following this theoretical reasoning, the study proposes the following hypothesis:

*H3:* Positive psychological capital mediates the relationship between job crafting and professional identity among primary and secondary school physical education teachers.

### 2.4 The relationship among job crafting, work stress, positive psychological capital, and professional identity

Research indicates that job crafting, as a proactive behavioral adjustment strategy, positively influences individuals’ professional identity (Uen et al., 2021). Job crafting is closely tied to individuals’ psychological resources. Other studies have found that teachers who proactively adjust their work tasks and relational resources are more likely to experience a sense of meaning and autonomy, thereby reducing work stress and accumulating positive psychological capital (Tims et al., 2016). According to the job demands-resources (JD-R) model, PE teachers exposed to prolonged high stress are more prone to emotional exhaustion, which in turn weakens professional identity (Alsalhe et al., 2021). Positive psychological capital, as a key internal resource, can buffer the negative effects of work stress and promote teachers’ positive evaluation of and emotional attachment to their profession. Building on the above theoretical framework, this study proposes that job crafting influences professional identity through a chain path of “reducing work stress – associated with positive psychological capital.” Specifically, as a form of resource acquisition and resource protection, job crafting helps PE teachers proactively design teaching tasks (e.g., innovating lesson content), expand social support networks (e.g., strengthening collaboration with colleagues and parents), and reframe work cognitions (e.g., re-interpreting the value of physical education for students’ holistic development). On the one hand, these behaviors are associated with lower work stress that co-occurs with role ambiguity, task overload, or insufficient resources. On the other hand, the sense of control and accomplishment derived from job crafting gradually builds up positive psychological capital, including self-efficacy, hope, optimism, and resilience. Increased positive psychological capital further associated with teachers’ ability to cope with work stress, enabling them to maintain a positive mindset when facing professional challenges, thereby indirectly mitigating the negative effects of work stress. At the same time, reduced work stress and associated with positive psychological capital reinforce each other: lower work stress provides a safe environment for accumulating positive psychological capital, and higher positive psychological capital helps teachers manage stressors more effectively, creating a virtuous cycle. Ultimately, this dual positive effect jointly strengthens PE teachers’ sense of professional identity – they are more likely to see themselves as competent in the PE teacher role, more willing to commit to the profession over the long term, and more able to derive greater value and meaning from their work. Based on the above reasoning, this study proposes the following hypothesis:

*H4:* Work stress and positive psychological capital play a chain mediating role between job crafting and professional identity among primary and secondary school physical education teachers.

To better integrate the theoretical perspectives, we anchor our chain mediation model primarily in the job demands-resources (JD-R) model and conservation of resources theory. Within this integrated framework, job crafting functions as a personal resource that reduces work stress (resource protection) and builds positive psychological capital (resource accumulation). Complementary insights from self-determination theory (autonomy, competence, relatedness) and positive organizational behavior theory (psychological capital as a developable state) are naturally incorporated rather than treated as separate parallel frameworks. This unified perspective allows us to propose a chain mediation model in which job crafting is associated with professional identity sequentially through reduced work stress and increased positive psychological capital.

**Figure 1:**
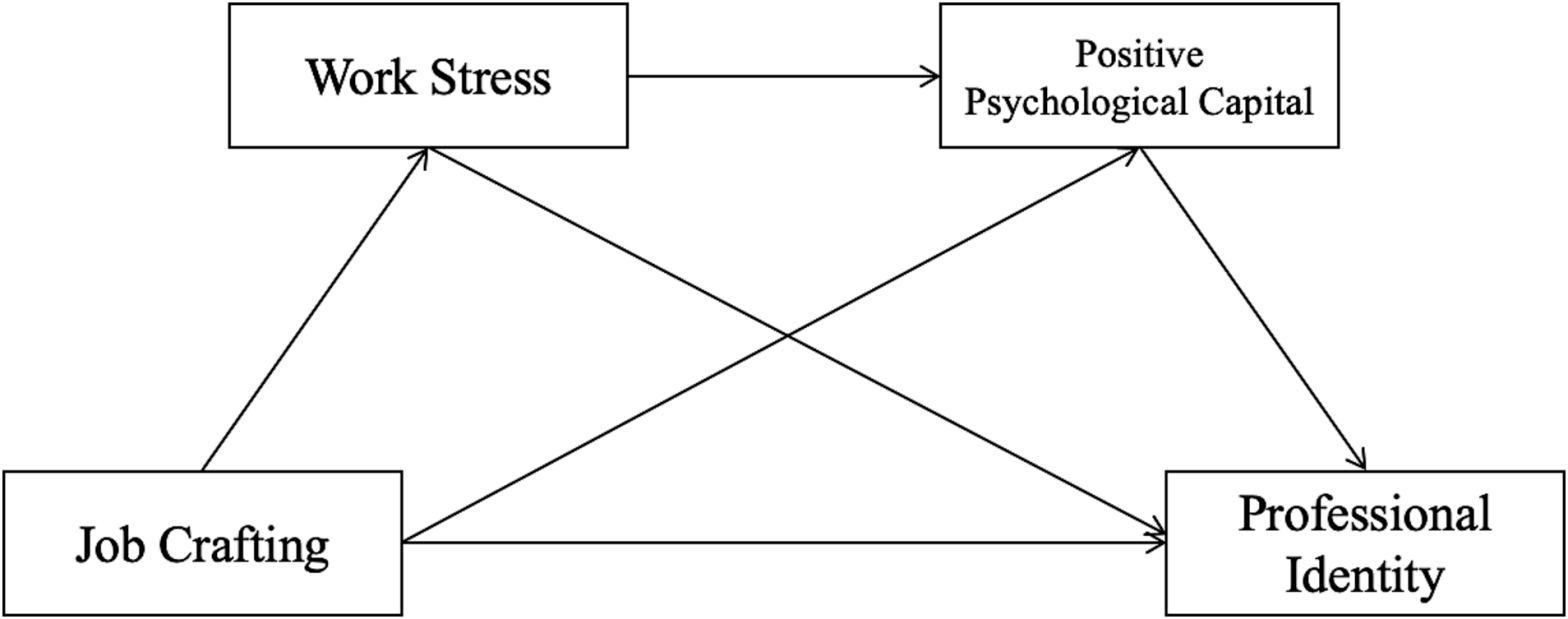
Chain mediation model: job crafting and professional identity.

Based on the proposed conceptual framework (Figure 1), the following hypotheses are put forward:

1. Job crafting is positively associated with professional identity among primary and secondary school PE teachers.
2. Work stress mediates the relationship between job crafting and professional identity.
3. Positive psychological capital mediates the relationship between job crafting and professional identity.
4. Work stress and positive psychological capital play a chain mediating role between job crafting and professional identity.

These hypotheses are grounded in the integrated theoretical framework described above. Testing this chain mediation model will help deepen our understanding of the psychological mechanisms linking job crafting to professional identity among primary and secondary school PE teachers.

## 3 Research methodology

### 3.1 Research sample

Data were collected using a self-administered questionnaire developed based on existing literature and validated instruments. The questionnaire assessed PE teachers’ job crafting behaviors, professional identity, work stress, and positive psychological capital. Since all participants were Chinese primary and secondary school PE teachers, the questionnaire was administered in Chinese. All scales had been translated into Chinese and validated among Chinese primary and secondary school teachers, showing satisfactory internal consistency and validity. The survey was conducted from April 12 to April 19, 2026, targeting primary and secondary school PE teachers from various provinces in China. A stratified cluster sampling method was adopted. First, China was divided into six geographical regions (East, South, North, Southwest, Central, Northwest). One or two provinces were randomly selected from each region, resulting in 10 provinces: Guangdong, Jiangsu, Zhejiang, Shandong, Henan, Sichuan, Hubei, Hebei, Hunan, and Shaanxi. Within each selected province, we randomly selected three prefecture—level cities as the primary sampling units. In each city, we randomly selected two schools (one primary, one secondary), and all PE teachers within the selected schools were invited to participate. This procedure yielded a total of 60 schools (10 provinces × 3 cities × 2 schools). The questionnaire was distributed online via the “Wenjuanxing” platform, with supervision from trained research assistants. Before data collection, all assistants familiarized themselves with the questionnaire structure and instructions, and briefed participants on key considerations before they started responding. Participation was entirely voluntary. A total of 900 questionnaires were distributed. After data cleaning, 850 valid questionnaires were retained (effective response rate 94.44%). Regarding sample size adequacy, with 51 scale items, the participant-to-item ratio of approximately 16.7:1 exceeds the commonly recommended 5:1 to 10:1 threshold (Costello and Osborne, 2005), and the absolute sample size of 850 is considered “very good” (Comrey and Lee, 2013). Moreover, for bias-corrected bootstrap mediation analysis, a minimum of 500 cases is recommended (Fritz and MacKinnon, 2007); our sample meets this requirement. To ensure data quality, the research team screened and excluded invalid responses based on the following criteria:

Inclusion criteria were: (1) currently working full-time as a PE teacher in a primary or secondary school in China; (2) at least one semester of teaching experience at that level; (3) provided voluntary informed consent.

Exclusion criteria included: (1) completing the questionnaire in less than 5 min (the median completion time from a pilot study was 10 min and 20 s); (2) evidence of patterned responses (e.g., selecting the same option consecutively); (3) failing an embedded attention check (“Please select ‘moderately agree’ or ‘strongly agree’ for this item”); (4) reporting implausible years of teaching or school information (e.g., teaching for over 50 years in a way inconsistent with reality).

After applying these criteria, 850 valid questionnaires were obtained, yielding an effective response rate of 94.44%. Among the valid sample, 50% were male (*n* = 425) and 50% female (*n* = 425), with a mean age of 34.75 ± 8.42 years.

### 3.2 Scale design

Job crafting was assessed using the Job Crafting Scale developed by Leana et al. (2009), which is grounded in the theoretical framework of job crafting proposed by Wrzesniewski and Dutton (2001). The scale is unidimensional and consists of four items rated on a 5-point Likert scale from 1 (“strongly disagree”) to 5 (“strongly agree”), measuring individuals’ proactive adjustments to their work approaches, such as introducing new methods to improve work, optimizing inefficient procedures, modifying ways of working to reduce difficulty, and rearranging equipment or furniture in the work environment. Higher scores indicate a stronger tendency to engage in job crafting. In this study, the scale showed good internal consistency, with a Cronbach’s alpha of 0.801.

#### 3.2.1 Professional identity

Professional identity was measured using the Primary and Secondary School Teachers’ Professional Identity Scale developed by Wei et al. (2013). The scale consists of 18 items rated on a 5-point Likert scale from 1 (“strongly disagree”) to 5 (“strongly agree”), covering four dimensions: role values (6 items), occupational behavior tendency (5 items), occupational values (4 items), and sense of professional belonging (3 items). The total score is obtained by summing the item scores across all dimensions, with higher scores indicating a stronger sense of professional identity. In this study, the scale demonstrated excellent internal consistency, with Cronbach’s alpha coefficients of 0.908 for the full scale, and 0.848, 0.787, 0.769, and 0.640 for the four dimensions, respectively.

#### 3.2.2 Work stress

Work stress was assessed using the Occupational Stress Scale developed by Motowidlo et al. (1986). The scale is unidimensional and consists of three items rated on a 5-point Likert scale from 1 (“strongly disagree”) to 5 (“strongly agree”), measuring individuals’ subjective perception of work stress. Example items include “My job is extremely stressful,” “There is seldom a stress-free moment in my work,” and “I feel a great deal of stress in my profession.” Higher scores indicate greater perceived work stress. In this study, the scale showed good internal consistency, with a Cronbach’s alpha of 0.784.

#### 3.2.3 Positive psychological capital

Positive psychological capital was measured using the Positive Psychological Capital Questionnaire developed by Kuo et al. (2010), which is grounded in the theoretical framework proposed by Luthans et al. (2006). The questionnaire consists of 26 items rated on a 7-point Likert scale, covering four dimensions: self-efficacy (7 items), resilience (7 items), hope (6 items), and optimism (6 items). Higher scores indicate higher levels of positive psychological capital. In this study, the questionnaire demonstrated excellent internal consistency, with Cronbach’s alpha coefficients of 0.939 for the full scale, and 0.871, 0.871, 0.858, and 0.852 for the four dimensions, respectively.

### 3.3 Statistical processing

This study adopted a quantitative approach, drawing on cross-sectional survey data to examine how job crafting, work stress, and positive psychological capital influence professional identity among primary and secondary school PE teachers. All statistical analyses were performed using SPSS 26.0, the PROCESS macro for SPSS (version 3.4; Model 6), and Excel 2021, following principles of transparency and reproducibility.

First, the collected questionnaires were screened for validity. Of the 900 distributed, 850 were valid, yielding an effective response rate of 94.44%. Second, common method bias was assessed using Harman’s single-factor test. The results showed that the first factor accounted for 28.47% of the total variance, below the recommended 40% threshold, indicating that common method bias was not a significant concern in this study (Podsakoff et al., 2003).

Descriptive statistics and Pearson correlation coefficients were computed for all study variables (job crafting, professional identity, work stress, and positive psychological capital). Hierarchical regression analysis was then performed to examine the direct relationships among these variables.

To test the proposed chain mediation model, the PROCESS macro (Model 6) was used with 5,000 bootstrap resamples to generate 95% bias-corrected confidence intervals for the indirect effects. This procedure quantified the total, direct, and indirect effects of job crafting on professional identity through work stress and positive psychological capital, assessed both independently and in sequence.

Confirmatory factor analysis (CFA). All scales used in this study have been previously validated in Chinese teacher samples via CFA (references). In the current sample, each scale achieved acceptable internal consistency. Therefore, we did not repeat CFA in this study.

## 4 Results

### 4.1 Sample characteristics

A total of 850 valid questionnaires were obtained, yielding an effective response rate of 94.44%. In terms of residential background, 312 participants (36.71%) came from urban areas, 368 (43.29%) from township areas, and 170 (20.00%) from rural areas. Regarding marital status, 589 participants (69.29%) were married, and 261 (30.71%) were single, divorced, or otherwise. As for monthly after-tax income, 54 participants (6.35%) reported below ¥3,000, 203 (23.88%) between ¥3,001 and ¥5,000, 356 (41.88%) between ¥5,001 and ¥7,000, 162 (19.06%) between ¥7,001 and ¥9,000, and 75 (8.82%) above ¥9,000. In terms of teaching experience, 189 participants (22.24%) had less than 5 years, 312 (36.71%) between 5 and 10 years, 226 (26.59%) between 11 and 15 years, 86 (10.12%) between 16 and 20 years, and 37 (4.35%) more than 20 years. The sample consisted of 425 male teachers (50.00%) and 425 female teachers (50.00%). The mean age of the participants was 34.75 years (SD = 8.42).

### 4.2 Common method bias test

Givn the self-report nature of the data collection, Harman’s single-factor test was used to assess common method bias. The unrotated exploratory factor analysis revealed five factors with eigenvalues greater than one. The first factor accounted for 28.47% of the total variance, below the recommended 40% threshold (Podsakoff et al., 2003). These results suggest that common method bias is unlikely to be a serious concern in this study.

### 4.3 Descriptive statistics and correlations for each variable

Descriptive statistics and Pearson correlation analyses were conducted for the four variables (job crafting, work stress, positive psychological capital, professional identity), as shown in Table 1. All variables were significantly intercorrelated: Job crafting was positively correlated with professional identity (r = 0.433, *p* < 0.01); job crafting was negatively correlated with work stress (r = −0.351, *p* < 0.01) and positively correlated with positive psychological capital (r = 0.459, *p* < 0.01); work stress was negatively correlated with positive psychological capital (r = −0.483, *p* < 0.01) and negatively correlated with professional identity (r = −0.453, *p* < 0.01); and positive psychological capital was positively correlated with professional identity (r = 0.517, *p* < 0.01).

**Table 1:** Descriptive statistics and correlations of variables.

Variable	Mean	SD	Job crafting	Work stress	Positive psychological capital	Professional identity
Job crafting	13.28	2.499	1			
Work stress	9.94	1.976	−0.351**	1		
Positive psychological capital	116.46	18.565	0.459**	−0.483**	1	
Professional identity	59.72	8.865	0.433**	−0.453**	0.517**	1

** indicates *p* < 0.01.

### 4.4 Testing of mediation effects

To examine the predictive effects of job crafting, work stress, and positive psychological capital on professional identity, a hierarchical regression analysis was conducted. Job crafting, work stress, and positive psychological capital were entered as independent variables, with professional identity as the dependent variable (Table 2). The results showed that: job crafting positively predicted professional identity (*β* = 0.433, t = 13.985, *p* < 0.001); work stress negatively predicted professional identity (β = −0.453, t = 14.778, *p* < 0.001); and positive psychological capital positively predicted professional identity (β = 0.517, t = 17.573, *p* < 0.001).

**Table 2:** R^2^adj denotes the adjusted R^2^.

Variable	Professional identity			
	β	T	F	R^2^adj
Job crafting	0.433	13.985***	195.570	0.186
Work stress	−0.453	−14.778***	218.375	0.204
Positive psychological capital	0.517	17.573***	308.814	0.266

***indicates *p* < 0.001.

This study examined the chain mediating effects of work stress and positive psychological capital on the relationship between job crafting and professional identity, with job crafting as the independent variable, professional identity as the dependent variable, and work stress and positive psychological capital as mediators, after controlling for demographic variables (e.g., gender, age). Analyses were performed using the PROCESS macro (version 3.4, Model 6) for SPSS 26.0 to test whether job crafting exerted significant mediating and chain mediating effects on professional identity. Hypothesis testing was conducted with PROCESS Model 6, applying 5,000 bootstrap resamples and 95% confidence intervals (CIs) to assess the chain mediation effects.

As shown in Table 3 mediation effect size and effect magnitude, job crafting had a significant positive effect on professional identity among primary and secondary school PE teachers (β = 1.536, *p* < 0.001; 95% CI [1.320, 1.751]). This indicates that higher job crafting scores predict a stronger sense of professional identity, supporting Hypothesis 1. The relationship between job crafting and professional identity was mediated by work stress (*p* < 0.001; 95% CI [0.197, 0.384]), suggesting that greater job crafting associated with professional identity by reducing work stress, thereby supporting Hypothesis 2. Positive psychological capital also mediated the effect of job crafting on professional identity (*p* < 0.001; 95% CI [0.264, 0.469]), meaning job crafting boosts professional identity by increasing positive psychological capital, which confirms Hypothesis 3. Finally, work stress and positive psychological capital chainly mediated the relationship (*p* < 0.001; 95% CI [0.100, 0.188]), demonstrating that job crafting raises professional identity through the sequential pathway of reducing work stress and then increasing positive psychological capital, thus confirming Hypothesis 4.

**Table 3:** Mediation effect size and effect magnitude.

Path	Effect	Boot SE	Boot LLC	Boot ULCI	Relative mediation effect
Total effect	1.536***	0.110	1.320	1.751	100%
Direct effect	0.746***	0.112	0.526	0.965	48.568%
Total indirect effect	0.790***	0.069	0.660	0.932	51.432%
indirect effect 1(WS)	0.286***	0.048	0.197	0.384	18.620%
indirect effect 2 (PPC)	0.363***	0.052	0.264	0.469	23.633%
indirect effect 3 (WS → PPC)	0.141***	0.022	0.100	0.188	9.180%

Indirect effect 1: Job Crafting → Work Stress → Professional Identity. Indirect effect 2: Job Crafting → Positive Psychological Capital → Professional Identity. Indirect effect 3: Job Crafting → Work Stress → Positive Psychological Capital → Professional Identity. ***indicates *p* < 0.001.

**Figure 2:**
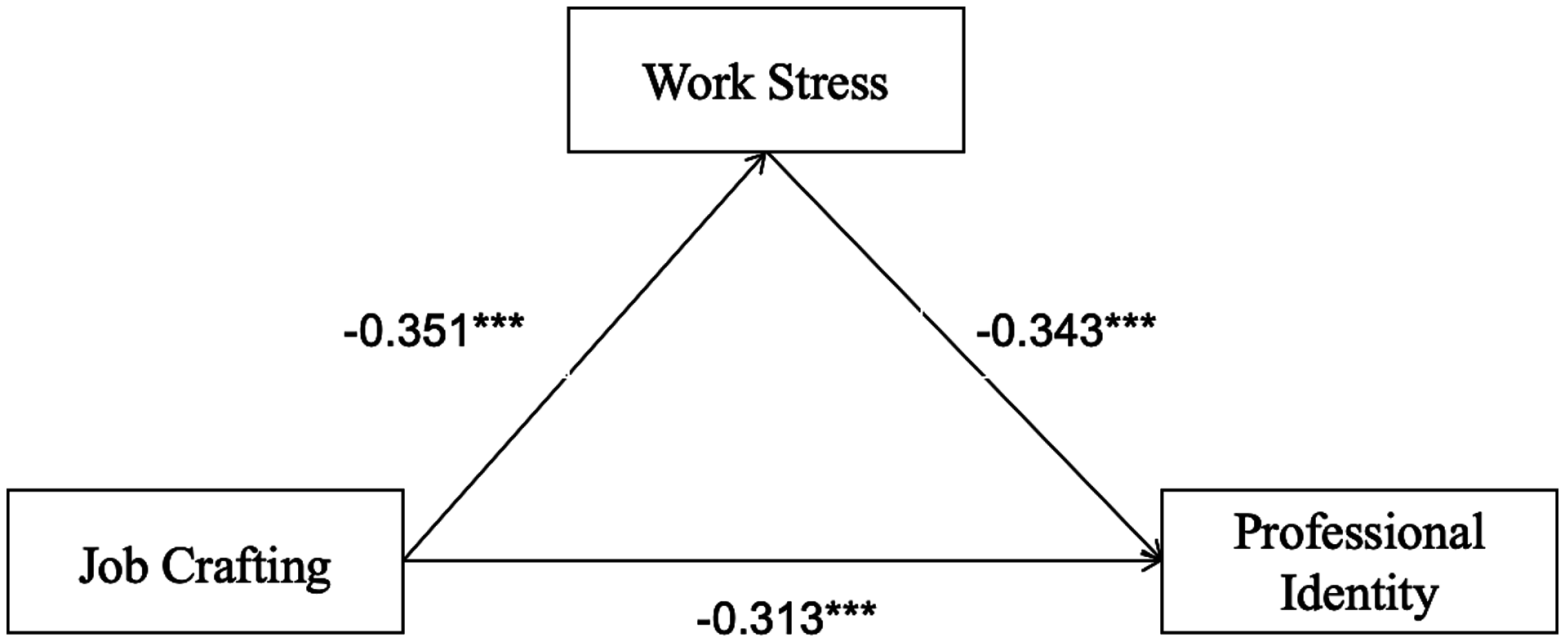
Mediation model of work stress between job crafting and professional identity. ***Denote statistical significance at the *p* < 0.001 level.

**Figure 3:**
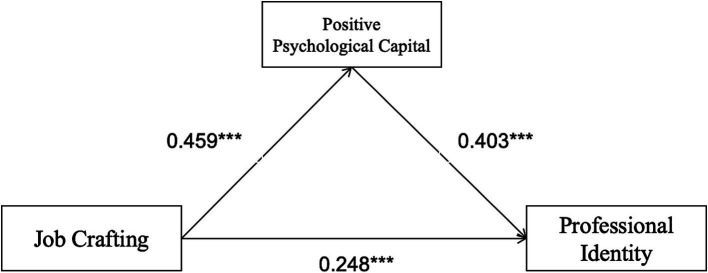
Mediation model of positive psychological capital between job crafting and professional identity. ***Denote statistical significance at the *p* < 0.001 level.

**Figure 4:**
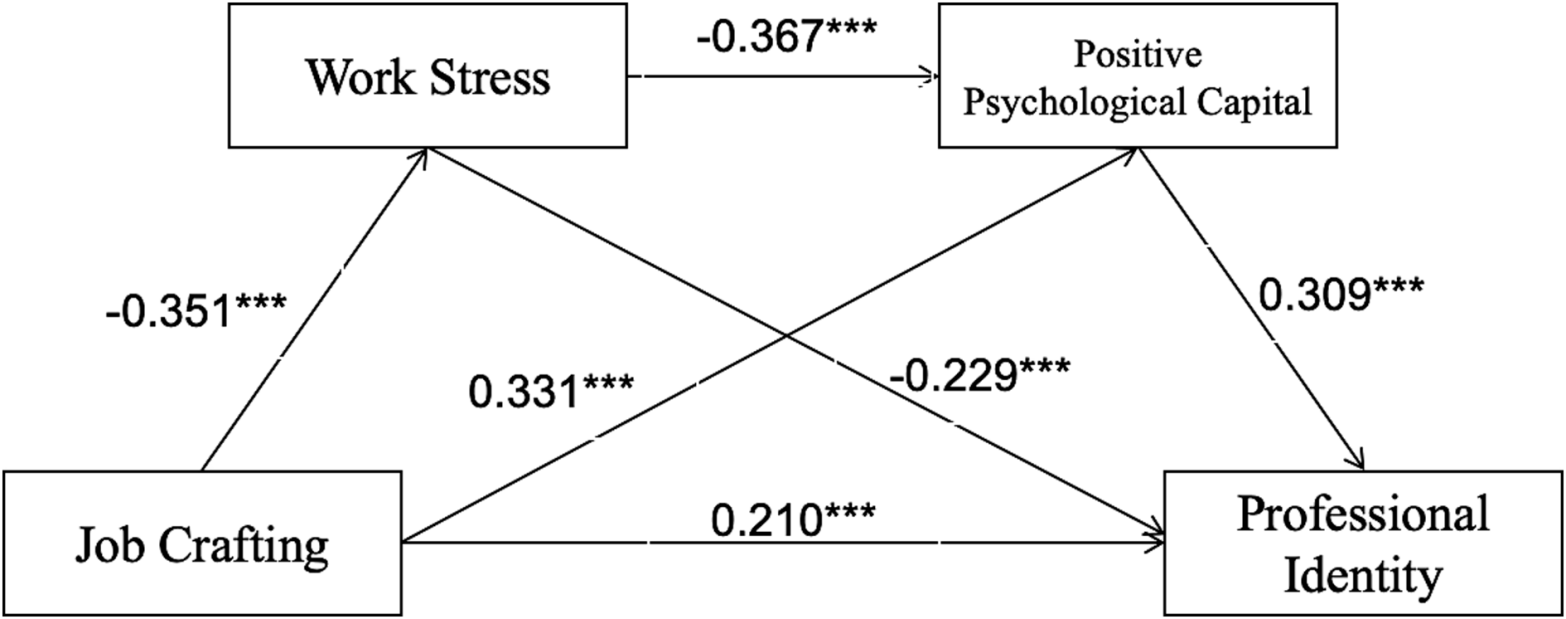
Chain mediation model of work stress and positive psychological capital between job crafting and professional identity. ***Denote statistical significance at the *p* < 0.001 level.

Moreover, the effect sizes for all three mediation pathways and the total effect were statistically significant (*p* < 0.001). The total effect was 1.536, with a direct effect of 0.746 (48.568% of the total effect) and an indirect effect of 0.790 (51.432% of the total effect). Among the mediation pathways: Path 1 (Job Crafting → Work Stress → Professional Identity) had an effect size of 0.286 (18.620% of the total effect); Path 2 (Job Crafting → Positive Psychological Capital → Professional Identity) had an effect size of 0.363 (23.633%); and Path 3 (Job Crafting → Work Stress → Positive Psychological Capital → Professional Identity) had an effect size of 0.141 (9.180%). The specific pathways are illustrated in Figures 2–4.

## 5 Discussion

Although Harman’s single-factor test (28.47% variance) suggested common method bias is not a serious concern, the cross-sectional self-report design may still inflate associations. Future research should use multi-source or longitudinal data to mitigate this bias.

### 5.1 Direct association between job crafting and professional identity

The results showed a significant positive correlation between job crafting and professional identity among primary and secondary school PE teachers (r = 0.433). After controlling for relevant variables and including mediators in the model, the direct link between job crafting and professional identity remained significant (*β* = 0.746, *p* < 0.001). Although previous studies have repeatedly shown that higher levels of job crafting are associated with higher professional identity, the present findings indicate that job crafting alone explains only a modest portion of the variance in professional identity (Wang et al., 2016). Thus, while the association is statistically significant, its practical magnitude calls for cautious interpretation. Even so, these results support the view that job crafting, as a proactive career development behavior, may help strengthen teachers’ professional identity (Zhai et al., 2025). This aligns with earlier findings that job crafting improves person-job fit, related to work meaning and autonomy, and thereby positively shapes occupational attitudes (Tims et al., 2013).

This finding suggests that job crafting not only correlates positively with professional identity at the behavioral level, but may also offer a more adaptive career development pathway for primary and secondary school PE teachers, potentially reducing their reliance on maladaptive coping strategies such as burnout or turnover intention. Unlike passively accepting work difficulties, job crafting provides these teachers with opportunities to proactively adjust their work tasks, relationships, and cognitions, thereby alleviating the professional identity crisis that might otherwise arise from role ambiguity or insufficient resources. The observed correlation (r = 0.433) reflects a moderate association between job crafting and professional identity. With the mediators taken into account, the direct effect remained significant (β = 0.746), indicating that mechanisms beyond work stress and positive psychological capital may also contribute to this relationship.

### 5.2 Mediating effect of work stress

Mediation analysis showed that work stress significantly mediated the relationship between job crafting and professional identity, with an indirect effect of 0.286, accounting for 18.620% of the total indirect effect. Although this indirect effect is considered small in absolute magnitude according to conventional guidelines (Cohen, 2013), its relative contribution underscores the critical role of work stress in explaining the link between job crafting and professional identity. This finding suggests that work stress serves as an important psychological mechanism through which job crafting relates to professional identity in this population.

Previous research has shown that job crafting is negatively related to work stress (Zhao et al., 2024), and work stress in turn is negatively related to professional identity (Zhang et al., 2024). The present study extends this evidence by showing that job crafting not only relates directly to professional identity but may also related to it indirectly by alleviating work stress among PE teachers, specifically through helping them redesign work tasks, expand social support networks, and reframe work meaning.

Against the unique policy backdrop of primary and secondary school physical education in China, this finding carries particularly strong practical implications. In recent years, the full implementation of the “Double Reduction” policy has brought profound changes to school physical education in China. On one hand, the policy explicitly requires ensuring students’ in-school physical activity time, leading to an increase in PE class hours (Liu and Wang, 2022); on the other hand, sports-related activities have become a key component of after-school services, requiring PE teachers to take on more extracurricular training, club supervision, and organization of after-school physical exercise (Liu and Mo, 2024). Meanwhile, PE teachers also face the issue of “structural shortage of teaching positions” – due to an overemphasis on core academic subjects, many schools have insufficient PE teacher positions, forcing existing PE teachers to shoulder an overloaded workload (Xu and Shi, 2025). The combination of these factors has led to a rapid increase in work stress among primary and secondary school PE teachers. The present study found that teachers with higher levels of job crafting can proactively adjust teaching content to match student interests, strengthen interdisciplinary collaboration, and optimize after-school service activity design, thereby alleviating to some extent the additional stress brought about by policy changes. By actively reframing the meaning of their work (e.g., viewing physical education as an important means of promoting students’ holistic development rather than merely a teaching task), they experience greater autonomy and competence, which in turn reduces stress reactions stemming from role ambiguity or excessive workload. Reduced work stress then fosters positive cognitions and emotional attachment to the profession, thereby associated with professional identity. Conversely, teachers lacking job crafting skills are more likely to fall into a passive coping mode, leading to persistently elevated stress and ultimately eroding professional identity.

### 5.3 Mediating effect of positive psychological capital

The current results show that positive psychological capital significantly mediated the relationship between job crafting and professional identity, with an indirect effect of 0.363 — the largest among the three mediation pathways — accounting for 23.633% of the total indirect effect. This finding aligns with positive organizational behavior theory, which holds that positive psychological capital (self-efficacy, hope, optimism, and resilience) positively predicts work attitudes and behaviors by strengthening individuals’ intrinsic motivation, coping capacity, and goal commitment (Luthans et al., 2007).

Job crafting is widely recognized as an effective strategy for promoting the accumulation of positive psychological capital (Luthans et al., 2016), and associated with positive psychological capital is a key pathway to fostering a stable professional identity among primary and secondary school PE teachers. When teachers gain more success experiences, positive feedback, and a sense of control through job crafting, their levels of self-efficacy, hope, optimism, and resilience increase accordingly, thereby strengthening their identification with the profession. The indirect effect of positive psychological capital (*β* = 0.363) accounted for 23.633% of the total indirect effect. This finding supports positive psychological capital as a core pathway linking job crafting to higher levels of professional identity.

### 5.4 Chain mediating effect of work stress and positive psychological capital

Chain mediation analysis showed that work stress and positive psychological capital formed a significant chain mediating pathway between job crafting and professional identity (indirect effect = 0.141, 95% CI [0.100, 0.188]). This finding suggests that job crafting may relate indirectly to professional identity among primary and secondary school PE teachers through a sequential mechanism of “reducing work stress → associated with positive psychological capital”.

This finding offers a more comprehensive view of the complex psychological mechanism linking job crafting to professional identity. Job crafting, as a resource-acquisition and resource-protection behavior, directly reduces work stress triggered by role ambiguity, task overload, or resource insufficiency on one hand (Bakker and Demerouti, 2017a); on the other hand, the sense of control and accomplishment derived from job crafting gradually accumulates into positive psychological capital (Apenbrink and Kuhlmann, 2025). Associated with positive psychological capital further strengthens teachers’ ability to cope with work stress, enabling them to maintain a positive mindset when facing professional challenges, thereby indirectly mitigating the negative effects of work stress. At the same time, reduced work stress and associated with positive psychological capital reinforce each other: lower work stress provides a safe environment for accumulating positive psychological capital (Bakker and Demerouti, 2017b), and higher positive psychological capital helps teachers manage stressors more effectively (Bidi et al., 2024), creating a virtuous cycle that jointly strengthens PE teachers’ sense of professional identity. This chain mediation aligns with theoretical frameworks that emphasize the interplay between work stress and positive psychological capital in shaping occupational attitudes (Grover et al., 2018). It also lays a foundation for future research to explore the psychological related to of job crafting on teachers’ career development from dynamic and process-oriented perspectives.

Notably, although all indirect effects were statistically significant, the effect sizes were relatively small. Several factors may account for this pattern. First, in mediation models involving chain pathways, the product of coefficients across multiple paths inevitably yields a value smaller than the direct effect or any single mediation effect (Kenny and Judd, 2014). Second, the total indirect effect (0.790) accounted for 51.432% of the total effect (1.536), indicating that work stress and positive psychological capital together make a substantial contribution to explaining the association between job crafting and professional identity. Third, from an educational administration perspective, even small effects can carry meaningful implications when applied to large populations of teachers (Panjeh et al., 2023). Given that low professional identity and frequent burnout among primary and secondary school PE teachers are widespread issues in China, interventions targeting work stress reduction and positive psychological capital enhancement—even if modest at the individual level—may still yield significant population-level benefits. Future research should further examine other mediating variables, such as organizational support and self-efficacy, to account for the remaining variance. Taken together, these findings provide empirical support for the above theoretical framework, showing that job crafting relates to professional identity through the sequential pathway of work stress (conservation of resources theory) and positive psychological capital (positive organizational behavior theory).

### 5.5 Research limitations and future prospects

Several limitations of this study should be acknowledged. First, the cross-sectional design precludes causal inference from the observed associations; the directional paths in our model are based on theoretical assumptions rather than empirical causality. Future research should test the proposed model using longitudinal designs or experimental interventions, for example, by tracking the long-term effects of job crafting interventions on teachers’ professional identity.

Second, all variables were measured using self-report instruments, which may be susceptible to recall bias and social desirability effects (Caputo, 2017). Although the scales showed adequate reliability and validity, future research could incorporate objective observations of job crafting behaviors (e.g., classroom video analysis) or multi-source assessments (e.g., peer ratings, principal feedback) to enhance measurement validity.

Third, although the sample covered primary and secondary school PE teachers from different school types (urban, township, rural) across 10 provinces in China with a balanced gender ratio, the findings may not be fully generalizable to teachers at other educational levels or to other occupational groups.

Moreover, the cross-sectional design precludes testing reverse causality (Savitz and Wellenius, 2023). While our theoretical model assumes that job crafting affects professional identity, the reverse may also hold: teachers with higher professional identity may be more proactive in job crafting, or those with lower work stress may be more willing to engage in job crafting. Future research should employ longitudinal or cross-lagged panel designs to examine these bidirectional relationships.

In addition, the sample included 50% male and 50% female participants, but no gender-specific analyses were conducted. Although the gender ratio was balanced, male and female teachers may differ in how they cope with work stress, accumulate positive psychological capital, and express professional identity (e.g., female teachers may focus more on relationship crafting, whereas male teachers may emphasize task crafting). Future research should examine whether gender moderates the proposed chain mediation model. Meanwhile, variables such as teaching experience, school type (urban vs. rural), and monthly income were not controlled for in the mediation analysis. While our primary focus was on psychological mechanisms, these demographic and occupational background variables may be related to the observed relationships (e.g., teachers with longer teaching experience may have accumulated higher positive psychological capital). Future research should include them as covariates to improve the precision of the estimated associations.

Moreover, this study did not distinguish between specific dimensions of job crafting (e.g., task crafting, relational crafting, cognitive crafting) or subdimensions of positive psychological capital (self-efficacy, hope, optimism, resilience). Different dimensions may play distinct roles between work stress and professional identity. For instance, relational crafting may reduce work stress more effectively than task crafting, and self-efficacy may predict professional identity more strongly than optimism. Future research should examine these dimensional differences in greater depth.

In addition, this study focused only on work stress and positive psychological capital as mediating variables, but other factors (e.g., organizational support, leadership style, collegial relationships) may also moderate or mediate the relationship between job crafting and professional identity. Future research could explore the roles of these variables, for example, whether school climate enhances the positive effects of job crafting.

Despite these limitations, this study enriches empirical research in the field by systematically clarifying how job crafting, work stress, and positive psychological capital influence professional identity among primary and secondary school PE teachers. The findings suggest that promoting job crafting not only directly enhances professional identity but may also indirectly strengthen it by reducing work stress and building positive psychological capital. This finding carries practical implications for educational administrators and schools in designing teacher development interventions. Combining job crafting training with stress management programs and positive psychological capital building may be an effective way to enhance professional identity among primary and secondary school PE teachers. For example, schools could organize job crafting workshops that encourage teachers to proactively adjust teaching tasks and interpersonal relationships, while also offering psychological capital enhancement training (e.g., self-efficacy building, resilience training). This may create a virtuous cycle that ultimately promotes the stability and professional growth of the PE teaching workforce.

## 6 Conclusion

This study systematically examined the relationship between job crafting and professional identity among primary and secondary school physical education teachers in China, with a particular focus on the mediating roles of work stress and positive psychological capital. The results showed a significant positive association between job crafting and professional identity, and this relationship was partially mediated by work stress and positive psychological capital through a significant chain mediating pathway.

Specifically, job crafting not only directly related to higher levels of professional identity but also did so indirectly through its negative association with work stress and positive association with positive psychological capital. Positive psychological capital emerged as the strongest mediator in the indirect pathway, highlighting its central role in this process. Work stress, acting as a risk factor, further clarified the psychological mechanism linking job crafting to professional identity. The chain mediation analysis further suggested that job crafting may influence teachers’ professional identity through a sequential process: first reducing work stress, then building positive psychological capital, and ultimately associated with professional identity.

By integrating behavioral and psychological perspectives, this study extends the existing research framework on the relationship between job crafting and professional identity, contributing to a deeper understanding of the mechanisms underlying the formation of professional identity among primary and secondary school PE teachers. At the practical level, the findings suggest that educational administrators and schools should consider the multifaceted role of job crafting in promoting teachers’ career development. Interventions that combine job crafting training with stress management programs and positive psychological capital building—particularly strategies that encourage teachers to proactively adjust work tasks, expand social support networks, and reframe professional cognitions—may be especially effective in associated with professional identity and supporting the stability of the primary and secondary school PE teaching workforce.

## Data Availability

The original contributions presented in the study are included in the article/Supplementary material, further inquiries can be directed to the corresponding author.
